# Corneal endothelial regeneration in human eyes using endothelium-free grafts

**DOI:** 10.1186/s12886-022-02260-x

**Published:** 2022-01-21

**Authors:** Lu-Yi Ying, Wen-Ya Qiu, Bing-Hong Wang, Ping Zhou, Bei Zhang, Yu-Feng Yao

**Affiliations:** 1 Department of Ophthalmology, Sir Run Run Shaw Hospital, Zhejiang University School of Medicine, 3 Qingchun Road East, Hangzhou 310016 Zhejiang Province, People’s Republic of China; 2 Key Laboratory for Corneal Diseases Research of Zhejiang Province, Zhejiang Province, People’s Republic of China

**Keywords:** Corneal endothelial regeneration, Penetrating keratoplasty, Corneal graft, Cryopreserved cornea

## Abstract

**Background:**

To report on corneal endothelial regeneration, graft clarity, and vision recovery when using endothelium-free grafts.

**Methods:**

We evaluated the donor’s cell viability using trypan blue staining and dual staining with calcein acetoxy methyl ester and ethidium homodimer-1. To preserve eyeball integrity, we performed therapeutic penetrating keratoplasty using cryopreserved donor tissue without endothelium on 195 consecutive patients who suffered from corneal perforation due to progressive primary corneal disease such as herpes simplex keratitis, fungal keratitis, ocular thermal burns, keratoconus, and phlyctenular keratoconjunctivitis. Of these, 18 eyes recovered corneal graft clarity and underwent periodic slit-lamp microscopy, A-scan pachymetry, and in vivo confocal microscopy to observe the clinical manifestations, variations in corneal thickness, and repopulation of the corneal endothelial cells on the donor grafts.

**Results:**

No viable cells were detected in the cryopreserved corneas. After the therapeutic penetrating keratoplasty, notable corneal graft edema was observed in all 18 eyes for 1–4 months, and no corneal endothelial cells were detected on the grafts during this period. Thereafter, we observed gradual and progressive regression and final resolution of the stromal edema, with complete recovery of corneal graft clarity. Through periodic confocal microscopy, we observed the corneal endothelium’s regenerating process, along with single cells bearing multiple nuclei and cell division-like morphology. The regenerated endothelium on the grafts reached a mean cell density of 991 cells/mm^2^. Remarkable vision rehabilitation was achieved in all 18 patients.

**Conclusions:**

We obtained conclusive evidence that host-derived endothelial cells can regenerate a new endothelium over the endothelium-free graft, which possesses normal functions for corneal clarity and vision recovery.

## Background

Corneal clarity crucially depends on a relatively dehydrated state for the stroma regulated by the endothelium, a densely packed, single layer of cells on the posterior surface of the cornea [1]. A corneal endothelium with a sufficient cell density helps maintain a normal hydration state and cornea clarity through the functions of a passive barrier and active ion transport. The endothelial cell density decreases slowly and linearly with age [2–5] and declines rapidly through accidental or surgical trauma [6, 7], corneal transplantation [8, 9], and diseases such as diabetes [10], glaucoma [11], and endothelial dystrophy [12, 13]. When the corneal endothelial cell density falls below a critical level [14, 15], the integrity of the endothelial barrier and pump functions is compromised, and corneal endothelial decompensation occurs, manifesting as epithelial bullae, stromal edema, corneal clouding, and severe vision impairment, which can only be reversed by the transplantation of donor cornea with a healthy endothelium [1, 16, 17].

Physically, human corneal endothelial cells lack regenerative capacity in vivo [18–20]. Endothelial wound healing in the defect area is accomplished by cell migration and enlargement [2, 20–22]. In contrast, ex vivo experiments have suggested that the periphery of the corneal endothelium has a higher regenerative capacity in vitro than the center [17, 23]. Most recently, experimental studies have shown that the corneal endothelium can regenerate in rat and rabbit eyes [24–27].

In our case series reported herein, patients with a variety of primary corneal diseases at a severe stage of perforation underwent therapeutic corneal transplantation to preserve the eyeball integrity [28]. We used cryopreserved donor corneas without endothelium for the therapeutic penetrating keratoplasty (PKP) [28]. Postoperatively, we observed that some of the grafts that initially presented notable edema and cloudy, had gradually and progressively improved; the stromal edema ultimately resolved in these grafts, followed by complete restoration of corneal clarity. Moreover, dynamic regeneration of a newly reconstructed endothelium on the grafts was substantiated through in vivo confocal microscopy at different time intervals. This study provides clear evidence that a new functional endothelium can be regenerated on endothelium-free corneal grafts in human eyes.

## Methods

### Cryopreservation of donor tissue

The donor corneas with primary endothelial deficiency or severe cell loss, unsuitable for optical PKP, were preserved at Zhejiang University Eye Bank in Sir Run Run Shaw Hospital (Zhejiang, China). The methods for preserving donor tissue under cryoconditions have been previously described [28–31]. In brief, the whole donor eyeball was cryopreserved at − 20 °C in a balanced salt solution containing 50 μg/ml of penicillin, 50 μg/ml of streptomycin, 100 μg/ml of neomycin, and 2.5 μg/ml of amphotericin B. The mean duration for the donor tissue cryopreservation before use was 9.5 ± 8.3 months.

After thawing, the corneal button was obtained from the donor eyeball. For transplantation, the anterior and posterior surfaces of the button were repeatedly scraped off using cotton swabs to remove epithelial and endothelial cell debris from the tissue and were aggressively washed using a balanced salt solution before grafting.

The corneal endothelial cell viability of the donor eyes after cryopreservation was assessed by trypan blue and dual staining of calcein acetoxy methyl ester and ethidium homodimer-1 [32–34], indicating that the residual endothelial cells were floating or accumulating as clusters. The individual cells exhibited shrinking and disruption into fragments, and, in particular areas of the posterior surface, there were no attached endothelial cells (data not shown).

### Patients

From May 1995 to April 2021, 195 consecutive patients underwent therapeutic PKP using cryopreserved donor tissue due to various primary corneal diseases in the severe stage. The PKP procedures were performed at the Department of Ophthalmology, Sir Run Run Shaw Hospital, Zhejiang University School of Medicine. We have previously reported the effectiveness of therapeutic PKP using cryopreserved donor corneal tissue for eradicating infection and preserving the eyeball integrity of eyes with severe fungal infection [28]. Our earlier study demonstrated that eyes with cryopreserved donor grafts exhibited persistent corneal cloudiness and edema even after six months or longer follow-up and that patients required a secondary PKP with a fresh donor to recover corneal clarity and achieve visual rehabilitation [35]. This case series, however, presented unexpected findings for 18 eyes from 18 of the 195 patients, in which the notably cloudiness and edema gradually subsided and the grafts became clear. The 18 patients completed a minimum of 12 months of postoperative follow-up and met this study’s inclusion criteria.

For the analysis, we respectively retrieved the patients’ demographic data (age/sex), primary corneal disease history, reason for undergoing therapeutic PKP, surgical history, slit-lamp microscopy and photography results, in vivo confocal microscopy results, and uncorrected and best corrected visual acuity readings at each visit (Table 1). We then analyzed the characteristics of the eyes whose grafts became clear and the eyes with persistently cloudy and edematous grafts.

**Table 1:** Characteristics of corneal endothelial regeneration on donor grafts after therapeutic penetrating keratoplasty

Patient No	Gender/Age (year)	Primary Disease	Reason requires T-PKP	Graft Characteristics		Changes of Graft after Surgery			Additional Ops	BCVA		Follow-up (Month)
				Centricity	Size (mm)	Earliest detectable ECs (Month)	Shift to complete clear (Month)	Final EC density (cells/mm^**2**^)		Pre-op	Last	
**1**	M/47	FK	Perforation & severe intraocular inflammation	centric	7.75	2.5	10	814	Phaco+IOL implant	LP	1.0	207
									Laser posterior capsulectomy			
**2**	M/35	Thermal Burn	Corneal perforation	Centric	7.75	1.5	2.5	1148	CLAU	HM	0.2	36
**3**	M/55	FK	Corneal perforation	Centric	8.0	3	5	875	none	FC	0.5	16
**4**	M/27	HSK	Corneal perforation	Eccentric	8.25	1	2	1531	none	HM	0.8	19
**5**	M/28	FK	Corneal perforation & severe intraocular inflammation	Centric	8.0	3	11	1340	none	HM	0.8	19
**6**	F/61	HSK	Large tear of DM, scheduled DALK shifted to temporary PK using cryopreserved tissue	Centric	7.75	4	7.5	819	none	FC	0.3	26
**7**	M /21	Keratoconus, Acute corneal hydrops	Large tear of DM, scheduled DALK shifted to temporary PK using cryopreserved tissue	Centric	8.0	1	6.5	1030	none	FC	0.9	37
**8**	F/22	Phlyctenular keratoconjunctivitis	Corneal perforation	Eccentric	7.5	3	6	848	none	0.4	0.7	20
**9**	M/65	HSK	Corneal perforation	Centric	8.0	1.5	10	853	ECCE+IOL	FC	0.2	12.5
**10**	M/22	Keratoconus	Large tear of DM, scheduled DALK shifted to temporary PK using cryopreserved tissue	Centric	7.75	1.5	2.5	782	none	0.1	0.6	38
**11**	M/48	Thermal Burn	Corneal perforation	Centric	8.0	3	7	1126	AMT + CLAU	HM	0.3	36
**12**	M/47	Thermal Burn	Corneal melting, perforation, prolapse of intraocular contents	Eccentric involving partial limbal area	7.0	2	6.5	939	AMT + CLAU	LP	0.2	24
**13**	M/48	HSK	Corneal perforation	Eccentric	7.5	3	8	1208	non	LP	0.5	168
**14**	M/16	Phlyctenular keratoconjunctivitis	Large tear of DM, scheduled DALK shifted to temporary PK using cryopreserved tissue	Centric	8.0	1	3	873	none	0.3	0.7	36
**15**	M/25	Keratoconus, Acute corneal hydrops	Large tear of DM, scheduled DALK shifted to temporary PK using cryopreserved tissue	Centric	7.5	3	5	882	none	FC	0.8	30
**16**	F/18	Trauma, Corneal laceration, Large tissue defect	Corneal perforation & laceration	Eccentric involving partial limbal area	9.5	1.5	5	859	CLAU	FC	0.2	72
**17**	F/15	Keratoconus, Acute corneal hydrops	Large tear of DM, scheduled DALK shifted to temporary PK using cryopreserved tissue	Centric	8.25	2	3.5	900	none	0.05	1	50.5
**18**	F/22	HSK	Large tear of DM, scheduled DALK shifted to temporary PK using cryopreserved tissue	Centric	8.0	3	5.5	1002	none	FC	0.1	20

*T-PKP* therapeutic penetrating keratoplasty, *Ops* operations, E*C* endothelial cell, *FK* fungal keratitis, *HSK* herpes simplex keratitis, *BCVA* best-corrected visual acuity, *CLAU* conjunctiva limbal autograft, *HM* hand motion, *LP* light perception, *AMT* amniotic membrane transplantation, *ALT* Allogeneic limbal transplantation. The size of the graft was 0.5 mm larger than the size of host trephination

### Surgical procedures and postoperative medication

The surgical procedure for therapeutic PKP has been previously described in detail [28]. For infectious keratitis with corneal perforation, the recipient cornea was removed using a corneal trephine to mark an incision, a diamond knife to deepen the incision, and corneal scissors to cut the tissue around the trephined margin. We performed dialysis of the anterior synechia, if necessary, through viscoelastic dialysis. The fibrinous membrane on the iris surface was carefully peeled off using forceps. The anterior chamber was aggressively irrigated with a balanced saline solution. An iridectomy was routinely performed for each patient before closing the host-graft junction using interrupted 10–0 nylon sutures.

For the eyes scheduled to undergo deep anterior lamellar keratoplasty (DALK) but whose Descemet membrane ruptured during the surgery, we used cryopreserved donor tissue as a temporary graft, waiting for a subsequent optical PKP when fresh donor was available [29–31].

The postoperative medication for the eyes with primarily infectious keratitis comprised continuous antimicrobial therapy until complete eradication of the infection was achieved, as judged clinically. For the eyes with primarily herpetic keratitis, the postoperative medication included prophylactic oral acyclovir and topical steroid eyedrops. For the other eyes with non-infectious corneal disease, the patients were prescribed 0.1% fluorometholone eyedrops four times daily, tapered, and withdrawn within 6 months.

## Results

### Patient characteristics

From May 1995 to April 2021, we consecutively performed 195 therapeutic PKPs on 195 patients using cryopreserved donor tissue. Of the 195 grafts, 18 (9.23%) regained postoperative corneal clarity. Of these 18 patients, 13 were male, five were female, and the mean age was 35 years (range 15–65 years). The primary disease included herpes simplex keratitis complicated with corneal perforation in three eyes, herpes simplex keratitis with failed DALK surgery due to Descemet membrane rupture in two eyes, ocular thermal burns complicated with corneal perforation in three eyes, keratoconus with failed DALK surgery due to Descemet membrane rupture in four eyes, fungal keratitis complicated with corneal perforation in three eyes, phlyctenular keratoconjunctivitis complicated with corneal perforation in one eye, phlyctenular keratoconjunctivitis with failed DALK surgery due to Descemet membrane rupture in one eye, and traumatic corneal laceration with a large tissue defect in one eye. The graft size ranged from 7.0 mm to 9.5 mm, with a mean of 7.92 mm. Thirteen of the 18 (72.2%) grafts were performed centrically, and 5 (27.8%) were performed eccentrically involving the partial limbus due to the original location of the lesion in the cornea (Table 1).

### The course and outcome of the corneal grafts that regained clarity

**Fig. 1:**
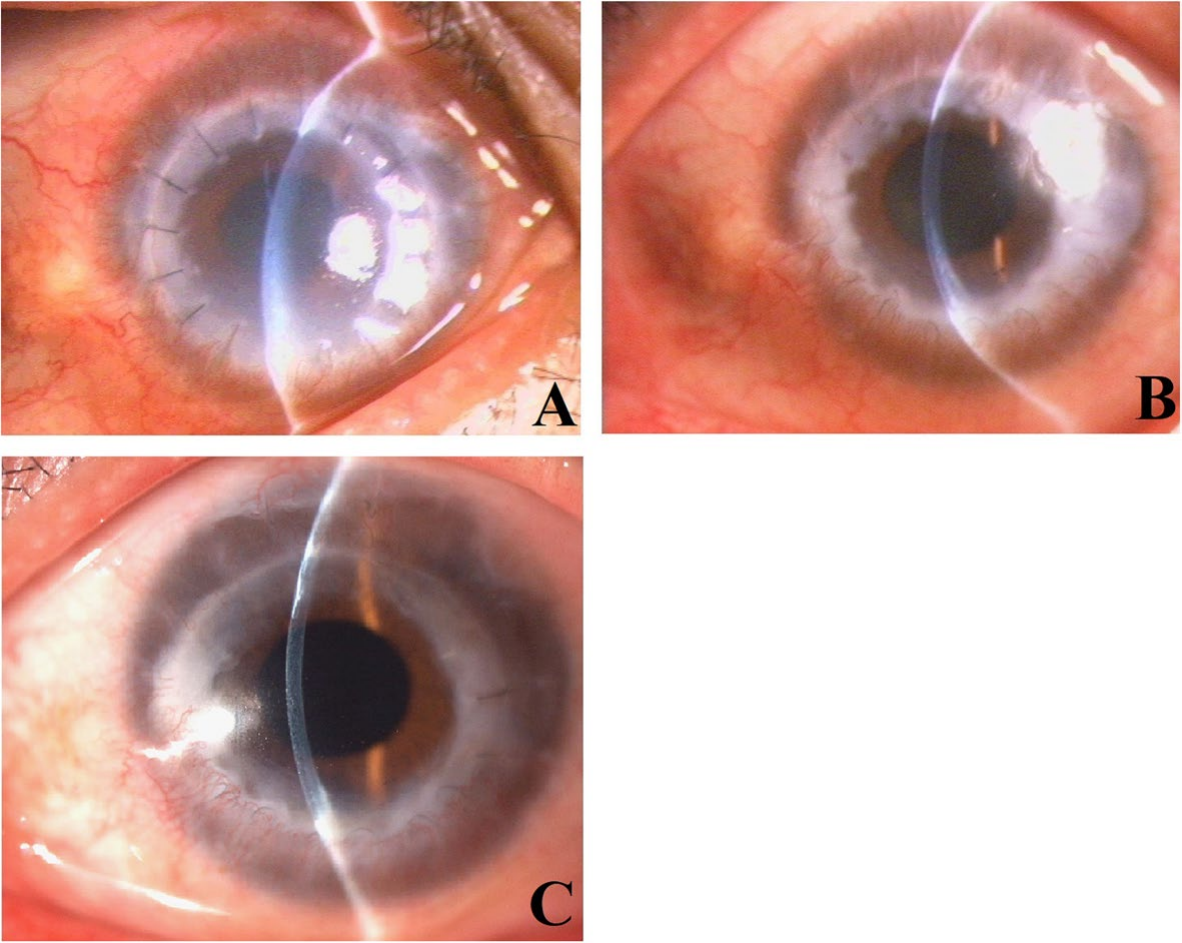
**A**: Representative case #1. Two months after the therapeutic penetrating keratoplasty using cryopreserved tissue, slit-lamp microscopy revealed significant edema and cloudiness in the corneal graft with mild conjunctival hyperemia. **B**: Representative case #1. Seven months after the therapeutic penetrating keratoplasty using cryopreserved tissue, slit-lamp microscopy showed that the graft had become comparatively clear but still retained a certain degree of edema, making the corneal graft obviously thicker than normal. **C**: Representative case #1. Ten months after the therapeutic penetrating keratoplasty using cryopreserved tissue, slit-lamp microscopy showed that the graft had become completely clear with normal corneal thickness

**Fig. 2:**
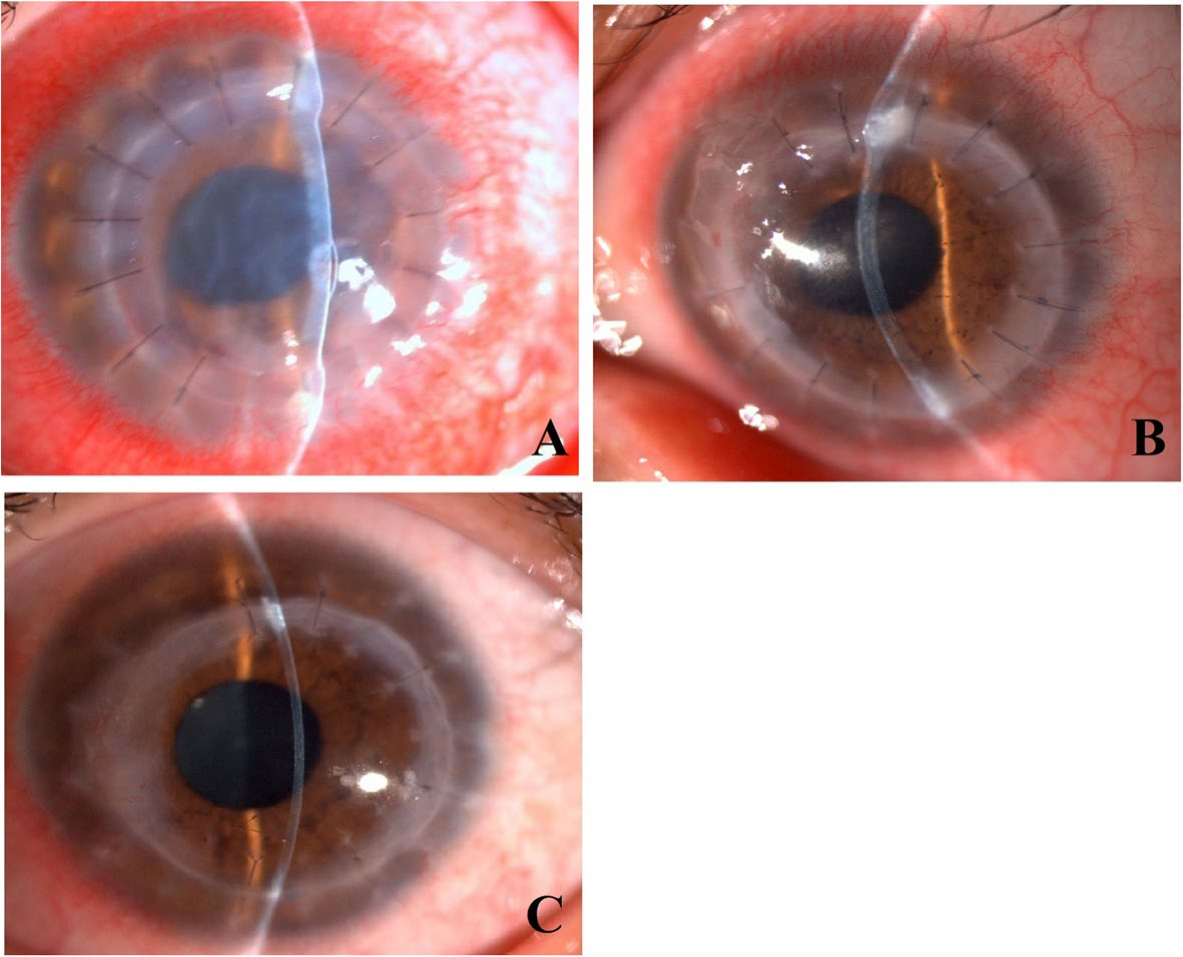
**A**: Representative case #5. One month after the therapeutic penetrating keratoplasty using cryopreserved tissue, slit-lamp microscopy revealed marked stromal edema and epithelial bullae coupled with corneal cloudiness of the graft and significant conjunctival hyperemia. **B**: Representative case #5. Three months after the therapeutic penetrating keratoplasty using cryopreserved tissue, the corneal graft had become almost clear with only mild edema in the stroma. Linear pigmented keratic precipitates on the posterior surface of the cornea can be clearly identified. The conjunctival hyperemia had also nearly resolved. **C**: Representative case #5. Eleven months after the therapeutic penetrating keratoplasty using cryopreserved tissue, the corneal graft became completely clear with no signs of inflammation, except for localized regional scarring around the graft-host junction

During the first postoperative stage, the corneal grafts were extremely edematous and markedly cloudy (Figs. 1A and 2A). The duration of the extreme edema and cloudiness varied among the 18 patients, ranging from approximately 1 month to 4 months, with a mean duration of 2 months. The graft edema subsequently subsided significantly, along with marked corneal thinning compared with previous manifestations, and the corneal clarity improved significantly, retaining however a certain degree of mild to moderate edema (Figs. 1B and 2B). The mild edema stage persisted for approximately 1–8 months. Thereafter, we observed complete resolution of the corneal edema, complete restoration of corneal clarity (Figs. 1C and Fig. 2C), and normalization of the corneal thickness of the grafts. Most of the patients reported sudden vision improvement at this stage. The corneal grafts remained clear in all cases throughout the follow-up period (mean follow-up, 48 months; range, 12.5–207 months).

**Fig. 3:**
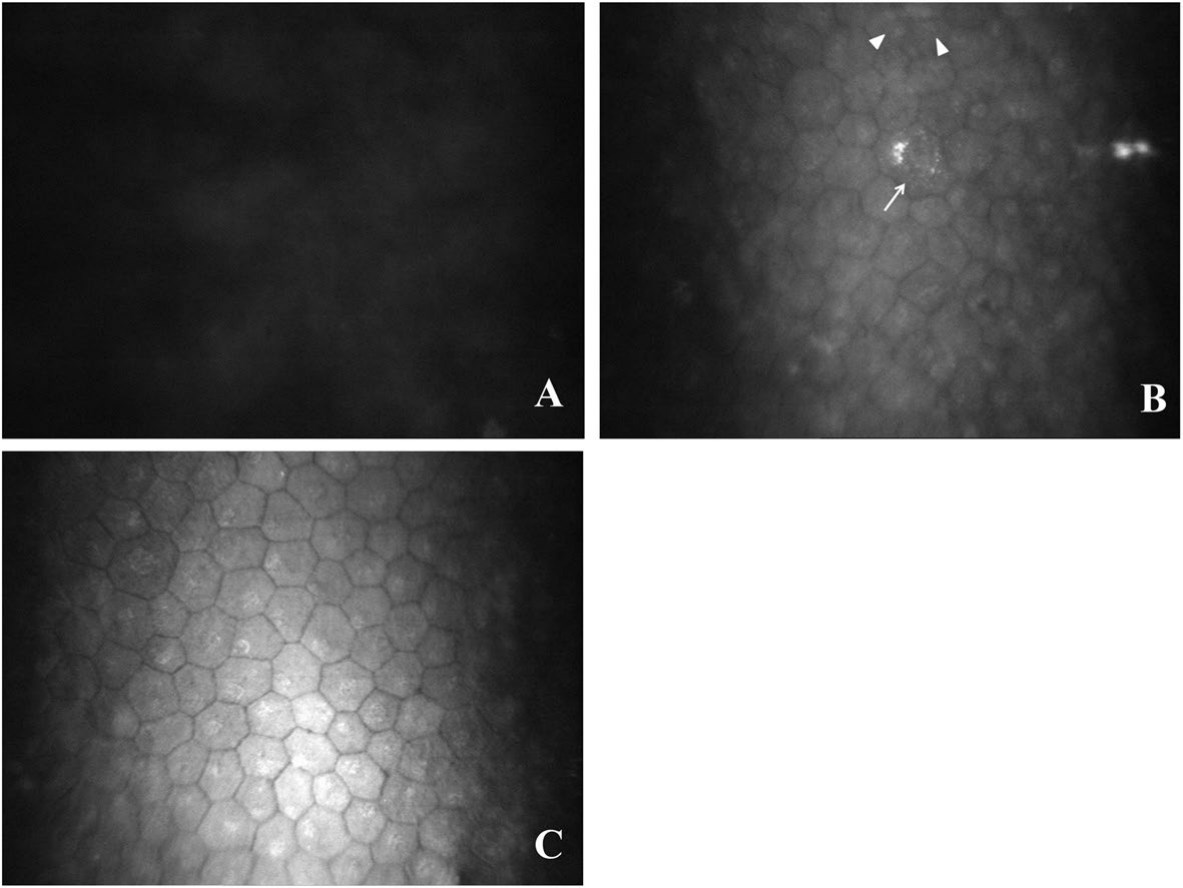
**A**: In vivo confocal microscopy of the corneal graft for representative case #1 at 2 months after therapeutic penetrating keratoplasty. Only a uniformly gray light reflection was observed, and no cells were detected on the posterior surface of the graft. **B**: In vivo confocal microscopy of the corneal graft for representative case #1 at 7 months after therapeutic penetrating keratoplasty. In vivo confocal microscopy revealed a formed endothelium with multiple nuclei in a single cell (triangle) and cell division morphology (arrow). The cell size and morphology varied markedly. **C**: In vivo confocal microscopy of the corneal graft for representative case #1 at 10 months after therapeutic penetrating keratoplasty. In vivo confocal microscopy revealed hexagonal and polygonal-shaped endothelial cells on the corneal graft, with a cell density of 1023 cells/mm^2^. The endothelial cells became relatively consistent in morphology and size

**Fig. 4:**
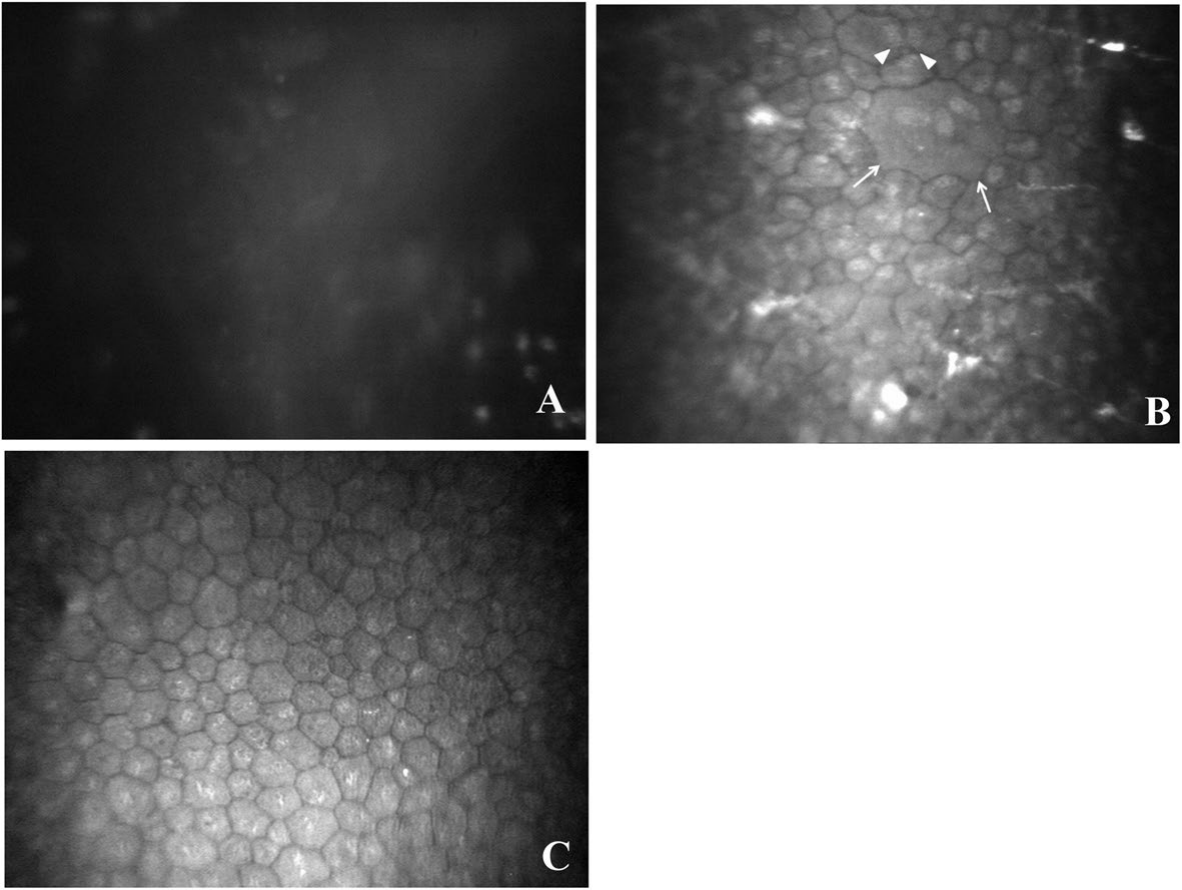
**A:** In vivo confocal microscopy of the corneal graft for representative case #5 at 1 month after the therapeutic penetrating keratoplasty. No corneal endothelial cells were detected on the posterior surface of the graft. **B**: In vivo confocal microscopy of the corneal graft for representative case #5 at 3 months after the therapeutic penetrating keratoplasty. A newly reconstructed corneal endothelium on the donor graft is observed, with markedly varied cell size and morphology, extraordinarily large cells containing multiple nuclei (triangle) and cell division (arrow). The cell density of the regenerated endothelium was 1186 cells/mm^2^ at this time. **C**: In vivo confocal microscopy of the corneal graft for representative case #5 at 11 months after the therapeutic penetrating keratoplasty. Corneal endothelial cell density of the endothelium on the graft became comparatively consistent in cell morphology and size. The cell density of the regenerated endothelium reached 1686 cells/mm^2^ at this time

Thorough confocal microscopy of the corneal grafts was performed in each case during each postoperative visit. During the stage where the corneal graft had extreme edema and cloudiness, in vivo confocal microscopy showed only uniformly gray light reflection with no cells detectable on the graft’s posterior surface (Figs. 3A and Fig. 4A). When the corneal edema notably subsided but remained mild, in vivo confocal microscopy detected endothelial cells, and the cell morphology and size varied markedly at this stage. In certain areas, the endothelial cells showed single cells containing multiple nuclei, ranging from 2 to 5 nuclei in a cell, with a seemingly cell division morphology (Figs. 3B and 4B). When the cornea became completely clear, the newly reconstructed endothelial cells became relatively consistent in morphology and size, reaching an mean cell density of 991 cells/mm^2^ (range, 782–1531 cells/mm^2^) (Figs. 3C and Fig. 4C). Throughout the mean follow-up of 48 months, the endothelial cell density in all grafts remained stable in all 18 cases.

### Characteristics of the eyes with the grafts that regained clarity and those with persistent cloudiness

The effectiveness of cryopreserved donor therapeutic PKP for eradicating infection and preserving the eyeball integrity of the eyes with severe fungal infection and tissue destruction has been reported elsewhere [28]. Since May 1995, we consecutively performed 195 therapeutic PKPs at our institute in 195 patients to treat severe corneal infection complicated with corneal perforation or non-infectious corneal perforation. Table 2 summarizes the characteristics of the eyes that presented graft clarity recovery and those with persistent graft cloudiness coupled with graft edema after therapeutic PKP. There were no significant differences in age and sex between the clear graft group and the persistently cloudy graft group. However, the primary diseases showed less severe infection and inflammation in the clear graft group than in the persistently cloudy graft group. The corneal perforation size in the clear graft group was significant smaller than in the persistently cloudy graft group (P < 0.001), and the graft size in the clear graft group was significant smaller than in the persistently cloudy graft group (P < 0.001). Lastly, the severity of the ocular inflammation and intraocular fibrous membrane for performing therapeutic PKP was significantly less severe in the clear graft group than in the persistently cloudy graft group (P < 0.001).

**Table 2:** Characteristics of the Eyes with Regaining Transparent Grafts and with Persistent Cloudy Grafts

		Groups			
		Grafts with Recovered Clear Cornea		Grafts with Persistent Cloudiness and Edema	
		Graft Number		Graft Number	
		18		177	
Gender		Male	Female	Male	Female
		13	5	113	64
Age (Average, Range)		37 ± 15 (16 ~ 65)	28 ± 19 (15 ~ 61)	38 ± 19 (20 ~ 66)	41 ± 11 (35 ~ 51)
Primary Disease	**Fungal Keratitis**	3	0	68	39
	**Bacterial Keratitis**	0	0	23	14
	**Acanthamoeba keratitis**	0	0	7	4
	**Herpes Simplex Keratitis**	3	2	9	5
	**Chemical or Thermal Burn**	3	0	6	2
	**Corneal Lacerative Trauma**	0	1	0	0
	**Phlyctenular Keratitis**	1	1	0	0
	**Keratoconus (Descemet Tearing in Performing DALK)**	3	1	0	0
Size of Corneal Perforation (mm)	**1 to 3**	12	5	11	5
	**3 to 6**	1	0	81	36
	**Over 6**	0	0	21	23
Severity of Ocular Inflammation & intraocular fibrous membrane	**Mild**	12	3	0	0
	**Moderate**	1	2	19	5
	**Severe**	0	0	94	59
Size of graft (mm)	**7.0 to 7.25**	1	0	0	0
	**7.5 to 7.75**	5	2	0	0
	**8.0 to 8.25**	7	2	0	0
	**8.5 to 8.75**	0	0	16	3
	**9.0 to 9.25**	0	0	76	51
	**Larger than 9.5**	0	1	21	10

### Case report

#### Case #1

*Fusarium solani* keratitis developed in the left eye of a 47-year-old male patient, which progressed into suppurative tissue necrosis and corneal perforation coupled with anterior chamber collapse despite aggressive antifungal medication. On May 22, 2003, the patient underwent therapeutic PKP using cryopreserved donor tissue to save the anatomical integrity of the eyeball and eradicate the fungal infection. The surgical procedure consisted of 7.5-mm-diameter-trephination and scissor cutting to remove the purulence and the entire infiltrate of the cornea. The anterior chamber was aggressively irrigated, and the fibrinoid membrane was carefully removed. An iridectomy at 5 o’clock was performed. A 7.75-mm cryopreserved corneal button was grafted using interrupted 10–0 nylon sutures. The anterior chamber was deepened using a balanced saline solution after suture closure of the donor-host junction.

The antifungal medication was continued postoperatively. Significant conjunctival hyperemia and fibrinous exudate in the anterior chamber were observed at one week after surgery. The inflammatory reactions including conjunctival hyperemia and fibrinous exudate in the anterior chamber subsequently regressed markedly, and completely resolved within 2 months after surgery. The systemic and topical antifungal drugs were withdrawn at the third month after surgery. The corneal graft demonstrated significant edema and cloudiness during the first 2 months after surgery (Fig. 1A). During the following 5 months after surgery, slit-lamp microscopy showed gradual regression of the corneal edema, but the thickness of the graft was still beyond the A-scan pachymetry measurement limitation. In vivo confocal microscopy showed only gray light reflection on the posterior surface of the graft (Fig. 3A). On December 17, 2003, seven months after the therapeutic PKP, we observed marked regression of the corneal edema with a corneal thickness of 655 μm at the center (Fig. 1B). In vivo confocal microscopy clearly revealed a formed endothelium with multiple nuclei in single cells and cell division morphology (Fig. 3B). On March 21, 2004, ten months after surgery, slit-lamp microscopy revealed complete resolution of the corneal edema and full restoration of corneal clarity in the graft (Fig. 1C). In vivo confocal microscopy clearly showed the presence of polygonal-shaped endothelial cells on the corneal graft, with a cell density of 1023 cells/mm^2^ (Fig. 3C). Due to the development of a cataract in the left eye, phacoemulsification surgery was performed on April 29, 2004, to remove the cataract, and an intraocular lens was implanted. Two months after the cataract surgery, in vivo confocal microscopy showed that the endothelial cell density have decreased to 868 cells/mm^2^. On August 18, 2020, 207 months after the therapeutic PKP, the corneal graft remained completely clear, and the endothelial cell density was 814 cells/mm^2^. The best corrected visual acuity of the left eye was 1.0.

#### Case # 5

A 28-year-old male patient developed suspected fungal keratitis in his left eye, showing corneal ulceration, and infiltrate with a feathery margin and corneal perforation. On December 24, 2004, the patient underwent therapeutic PKP with an 8.0-mm-diameter graft. The antifungal medication was continued postoperatively. On January 25, 2005, one month after surgery, slit-lamp microscopy showed moderate conjunctival hyperemia and marked edema in the corneal graft with a well-reconstructed anterior chamber in the left eye (Fig. 2A). Confocal microscopy detected no corneal endothelial cells of the graft during this visit (Fig. 4A). On March 23, 2005, three months after the therapeutic PKP, slit-lamp microscopy showed mild edema in the corneal graft with linear pigmented keratic precipitates on the posterior surface of the cornea, coupled with moderate conjunctival hyperemia in the left eye (Fig. 2B). In vivo confocal microscopy clearly showed the presence of polygonal-shaped corneal endothelial cells with multiple nuclei in single cells and seemingly cell division morphology, with a cell density of 1186 cells/mm^2^ (Fig. 4B). On November 7, 2005, 11 months after the therapeutic PKP, the corneal graft was completely clear, except for localized regional scarring around the graft-host junction (Fig. 2C). The corneal endothelial cell density reached 1686 cells/mm^2^, with comparative consistency in cell morphology and size (Fig. 4C). The best corrected visual acuity was 0.8. The corneal clarity and endothelial cell density of the graft remained stable through 19 months of follow-up.

## Discussion

Cryopreservation of corneal tissue at − 20 °C can result in a lack of viable epithelial cells, keratocytes and endothelial cells..

We previously demonstrated that cryopreserved donor corneas can be effectively used as substitutes in therapeutic PKP to help control and eradicate severe corneal infection and tectonically preserve the anatomical integrity of the eyeball [28]. Given that the donor button is devoid of endothelium, the corneal graft inevitably develops marked postoperative edema, as found in a previous study [28]. To recover corneal clarity and visual acuity, a secondary optical PKP with a healthy endothelial graft is required [28, 35]. Of the 195 consecutive patients in this study who underwent therapeutic PKP with a cryopreserved donor graft, however, 18 grafts in 18 eyes developed gradual and progressive regression of the edema and ultimately resolved the corneal edema, along with complete postoperative recovery of corneal clarity. This study clearly demonstrated that the recovery of corneal clarity in the graft was achieved by the regeneration of the corneal endothelium on the endothelial-free grafts with a comparatively high cell density, as confirmed by in vivo confocal microscopy. The periodic confocal microscopy check-ups also confirmed that a newly regenerated endothelium is most likely accomplished through cell proliferation rather than by cell migration. After the corneal edema regressed and the graft started to become relatively clear, confocal microscopy clearly showed that the endothelial cells appeared on the posterior surface of the graft, beginning with a few cells, gradually increasing in number, and ultimately arriving at a stable state. More importantly, the phenomena of multiple nuclei in a single cell and cell division-like morphology was clearly captured by in vivo confocal microscopy. The evidence shows that the endothelial formation was the result of cell proliferation rather than cell migration, which constructs a new layer of endothelium on the graft. The newly reconstructed endothelium subsequently achieves subsidence of the graft edema and restores clarity to the graft. Given that the graft lacks an endothelial layer and living cells, the endothelial cell proliferation on the graft can only be explained as having originated from the residual cells inside the host.

In Case #1, the regenerated corneal endothelial cells on the graft appeared to be tenacious enough to endure secondary phacoemulsification for cataract removal and intraocular lens implantation after more than 10 years of observation. The regenerated endothelium was shown to be stable in all grafts in all 18 cases, with a mean follow-up of 48 months.

Human corneal endothelial cells are generally thought to possess no proliferative capacity in vivo [18, 19, 36] and are difficult to establish in long-term cultures in vitro [37–39]. However, accumulated evidence indicates that several intrinsic [40–44] and extrinsic factors [45, 46] might help maintain the endothelium in a non-replicative state. The corneal endothelium in vivo does possess proliferative capacity but is arrested in the G1-phase of the cell cycle [14]. It is therefore reasonable to speculate that, when the non-replicative state is overcome, possibly contributing to a certain degree of inflammatory reaction in the eyes after therapeutic PKP, sufficient mitogenic stimulation factors are created in the anterior chamber to help the host’s residual endothelial cells to start proliferating and forming a new endothelium on the graft. The newly reconstructed endothelium on the graft exerts barrier and ion-pump functions and helps maintain the stroma’s hydration state, allowing the graft to regain its clarity.

We do not yet know the types of mitogenic stimulation factors and mechanisms involved in the regeneration of the endothelium on the endothelial-free grafts in our patients. The characteristics of the patients with endothelial cell regeneration on the graft can be summarized as follows: First, the inflammation varied among the eyes, ranging from originally infectious disease to non-inflammatory corneal disease prior to surgery. Second, postoperative inflammation appeared in all eyes due to the original disease and the surgery. Third, graft size ranged from 7.0 to 9.5 mmwith mean size of 8 mm. Fourth, the donor cornea had been cryopreserved and was devoid of cells including endothelial cells in the tissue; however, the Descemet membrane retained intact for transplantation.

In our case series, the earliest onset of endothelial cell growth, the time to the highest endothelial cell density, and the final endothelial cell density appear not to be significantly related to recipient age (at least not in terms of the younger the better) but was apparently associated with the primary preoperative disease and inflammation. It is worth noting that a young age and non-inflammatory disease did not have a positive association with endothelial regeneration. In contrast, preoperative infectious disease and a certain extent of preoperative and postoperative inflammation had a greater association with endothelial regeneration and a higher endothelial cell density. There is no doubt that the detailed mechanism of endothelial regeneration requires further study.

It is highly encouraging that the regenerated endothelium on the graft can reach a fairly high cell density that can sufficiently restore the comparatively dehydrated state of the stroma and recover the corneal clarity. The cell density of the newly reconstructed endothelium on the grafts can reach approximately 991 cells/mm^2^ on average. These findings might be helpful in developing new therapeutic approaches for treating patients with corneal endothelial decompensation. To further illustrate the mechanism involved in endothelial regeneration on endothelium-free corneal grafts, it might be necessary to first simulate the methods performed on this study’s patients using cryopreserved donor corneas to perform the therapeutic PKP on large animals or even in primates to determine the incidence of endothelial regeneration after such a transplantation. Using an animal model, we might be able to explore the detailed mechanisms with regard to cell factors that enable endothelial regeneration on the endothelium-free donor graft after cryopreservation. When the mechanism and method of in vivo endothelial regeneration on endothelium-free donor grafts become clear and reliable, we can reasonably speculate that the current optical PKP using endothelium-healthy donors will likely be replaced by endothelium-free cryopreserved donor corneas. This speculation is not only encouraged by the findings of our current case series but is also supported by several newly advanced approaches such as Descemet stripping only [47] and descemetorhexis combined with rho-kinase inhibitor [48] for treating Fuchs endothelial dystrophy. A recent study showed that the injection of cultured cells with a rho-kinase inhibitor can treat bullous keratopathy [49]. Taken together, endothelial-free grafting, Descemet stripping only and rho-kinase inhibitors can act as stimuli to help break through the arrested cell cycle phase and enable endothelial regrowth on the posterior corneal surface [50, 51].

Given that the endothelium can be regenerated on the donor graft, it is important to expand the donor source, and, given that the regenerated endothelium is derived from the host itself, there will be no immunoreactions targeting the donor endothelium and no graft failure caused by endothelial rejection after PKP [16, 52].

## Conclusion

Our study showed that host-derived endothelial cells can regenerate a new endothelium over an endothelium-free graft, which possesses normal functions for corneal clarity and vision recovery.

## Data Availability

All data generated or analysed during this study are included in this published article.

## Abbreviations

PKP: Penetrating keratoplasty
DALK: Deep anterior lamellar keratoplasty
